# Immunological Aspects in Late Phase of Living Donor Liver Transplant Patients: Usefulness of Monitoring Peripheral Blood CD4+ Adenosine Triphosphate Activity

**DOI:** 10.1155/2013/982163

**Published:** 2013-09-26

**Authors:** Shugo Mizuno, Yuichi Muraki, Kaname Nakatani, Akihiro Tanemura, Naohisa Kuriyama, Ichiro Ohsawa, Yoshinori Azumi, Masashi Kishiwada, Masanobu Usui, Hiroyuki Sakurai, Masami Tabata, Norihiko Yamamoto, Tomomi Yamada, Katsuya Shiraki, Yoshiyuki Takei, Tsutomu Nobori, Masahiro Okuda, Shuji Isaji

**Affiliations:** ^1^Department of Hepatobiliary Pancreatic and Transplant Surgery, Mie University School of Medicine, 2-174 Edobashi, Tsu, Mie 514-0001, Japan; ^2^Department of Pharmacy, Mie University School of Medicine, 2-174 Edobashi, Tsu, Mie 514-0001, Japan; ^3^Department of Molecular and Laboratory Medicine, Mie University School of Medicine, 2-174 Edobashi, Tsu, Mie 514-0001, Japan; ^4^Department of Gastroenterology and Hepatology, Mie University School of Medicine, 2-174 Edobashi, Tsu, Mie 514-0001, Japan; ^5^Translational Medical Science, Mie University School of Medicine, 2-174 Edobashi, Tsu, Mie 514-0001, Japan

## Abstract

*Aim*. To evaluate whether the combination of the peripheral blood CD4+ adenosine triphosphate activity (ATP) assay (ImmuKnow assay: IMK assay) and cytochrome P450 3A5 (CYP3A5) genotype assay is useful for monitoring of immunological aspects in the patient followup of more than one year after living donor liver transplantation (LDLT). *Methods*. Forty-nine patients, who underwent LDLT more than one year ago, were randomly screened by using IMK assay from January 2010 to December 2011, and the complete medical records of each patient were obtained. The CYP3A5 genotypes were examined in thirty-nine patients of them. *Results*. The mean ATP level of the IMK assay was significantly lower in the patients with infection including recurrence of hepatitis C (HCV) (*n* = 10) than in those without infection (*n* = 39): 185 versus 350 ng/mL (*P* < 0.001), while it was significantly higher in the patients with rejection (*n* = 4) than in those without rejection (*n* = 45): 663 versus 306 ng/mL (*P* < 0.001). The IMK assay showed favorable sensitivity/specificity for infection (0.909/0.842) as well as acute rejection (1.0/0.911). CYP3A5 genotypes in both recipient and donor did not affect incidence of infectious complications. *Conclusions*. In the late phase of LDLT patients, the IMK assay is very useful for monitoring immunological aspects including bacterial infection, recurrence of HCV, and rejection.

## 1. Introduction

In liver transplantation (LT), the introduction of a variety of immunosuppressive agents, the advances in surgical technique, and the short-term graft survival have been greatly improved in the last two decades. However, long-term graft survival remains unsatisfactory and one of the leading causes is the difficulty in the maintenance of an adequate level of immunosuppressive agents. Too little immunosuppressive status can lead to increasing the risks of acute and chronic rejection [1], whereas too much immunosuppression may cause malignant tumors, opportunistic infections, and drug toxicities [2, 3]. The most transplant centers assess the immunological status of the graft liver by measuring trough levels of calcineurin inhibitors (CIs) combined with laboratory data [4, 5], although neither of them is sensitive or specific for determining the current immunosuppressive status.

In 2002, the Food and Drug Administration approved an *in vitro* assay, the ImmuKnow (IMK) assay, which measures the ability of CD4+ T cells to respond to mitogenic stimulation by phytohemagglutinin-L *in vitro*. This assay is developed as an additional tool to adjust the immunosuppressive treatment [6–9], and this has a possibility to predict the mortality as well as the status of immunosuppressant agent condition in LT recipients [10]. However, little is known about the usefulness of this assay as a monitoring tool of immunological aspects in late phase of LT patients.

Tacrolimus, which is extensively used as an immunosuppressive drug in LT and has a narrow therapeutic window, is mainly metabolized by cytochrome P450 (CYP) 3A4 and CYP3A5 in the small intestine and the liver [6, 7], and CYP3A5 plays a key role in the pharmacokinetics of tacrolimus especially in LT patients [8, 9]. Although a previous study [10] reported that the CYP3A5 genotype in both recipients and donors affected infectious complications within 6 months following living donor LT (LDLT), the influence of CYP3A5 genotype on late allograft dysfunction remains unclear.

We hypothesized that the IMK assay can be useful for monitoring of immunological aspects in the late phase after LDLT, and that the CYP3A5 genotypes in both recipients and donors affect late allograft dysfunction. In this study, we focused on the patients more than one year after LDLT. We decided to use the IMK assay for studying the frequency of infectious complications in patients with the results of this assay below a target immunological response zone and the frequency of rejection in patients with IMK assay above a target zone. We also decided to study the influence of the combination of donor's and recipient's CYP3A5 genotype on infectious complications and/or rejection.

## 2. Patients and Methods

### 2.1. Patients

In LDLT patients who were treated at the Mie University Hospital between March 2002 and December 2011, 89 patients, who had been followed as outpatients for more than one year ago after LDLT, were candidates for this study. The inclusion criteria were LDLT patients, who happened to be treated either as inpatients or who had return visits to the clinic on a Thursday during the period from January 2010 to December 2011. This study was retrospective cohort study. A total of 49 LDLT patients were screened using the IMK assay and were observed clinically. The only exclusion criterion was if the patient was followed at other center than the Mie University Hospital. The complete medical records of each patient were obtained. Acute cellular rejection was defined by the 9-point Banff rejection activity index [11] as mild, moderate, or severe based on a liver biopsy at the time of undergoing the IMK assay when rejection was suspected clinically.  Table 1 shows patient characteristics in this study. The CYP3A5 genotypes were examined in 39 patients who underwent LDLT after September 2005 [12]. This study (IMK assay and CYP3A5 genotypes) was approved by institutional review board of Mie University Hospital and patients' consent was obtained.

### 2.2. ImmuKnow (IMK) Assay

Blood samples were collected in sodium heparin tubes, and the intracellular adenosine triphosphate activity (ATP) level was measured. Blood samples were processed on the day of sample collection. Briefly, 250 *µ*L of anticoagulated whole blood was diluted with the provided sample diluent to make a final volume of 1000 *µ*L. Samples were added to wells of a 96-well plate and incubated from 15 to 18 h with phytohemagglutinin at 37°C and 5% CO_2_ atmosphere. After enrichment of CD4+ T cells by addition of magnetic particles coated with an anti-human CD4 monoclonal antibody (Dynabeads, Dynal, Oslo, Norway), cells were washed and lysed to release intracellular ATP. Released ATP was measured with a luciferin/luciferase assay in a luminometer. The patient's level of immune response was expressed as the amount of ATP (ng/mL). According to a previous report [8], we used the cutoff ATP level of 225 ng/mL for identifying risk of infection and 525 ng/mL for rejection, and we defined a target immunological response zone ranging from 226 to 525 ng/mL.

### 2.3. Immunosuppression

The immunosuppression protocol consisted of tacrolimus and low-dose steroids. The target whole-blood trough level for tacrolimus was from 10 to 12 ng/mL during the first 2 weeks, approximately 10 ng/mL thereafter, and from 5 to 10 ng/mL from the second month after LDLT. Methylprednisolone (1 mg/kg per day, intravenously) was given on postoperative days 1 to 3, followed by 0.5 mg/kg per day on postoperative days 4 to 6. Steroid administration was then switched to oral prednisolone (0.3 mg/kg per day) on postoperative day 7, and the dose was reduced to 0.1 mg/kg per day at 1 month after LDLT. If their liver function was stable, recipients were weaned off steroids at 3 to 6 months after LDLT.

### 2.4. Evaluation of Tacrolimus Blood Concentration and Concentration/Dose (C/D) Ratio

We collected 1 mL of blood treated with EDTA for anticoagulation at 12 h after the previous dose, and the tacrolimus blood concentration was then measured by using a semiautomated microparticle enzyme immunoassay (IMx, Abbott Co., Ltd., Tokyo, Japan). The daily dose of tacrolimus was recorded and its weight-adjusted dosage (mg/kg per day) was calculated. Then, the measured blood tacrolimus concentration was normalized by the corresponding dose per body weight 24 h before blood sampling to obtain the concentration/dose (C/D) ratio, which was then used for estimating the tacrolimus dose needed to achieve the target trough concentration.

### 2.5. Genotyping of Cytochrome P450 3A5

Genomic DNA was isolated by using the QIAamp Blood Kit (Qiagen, Hilden, Germany). A fragment containing the A6986G polymorphism was amplified as follows. The PCR samples contained 10× PCR buffer, 2 mM deoxyribonucleoside triphosphate, 0.1 mM primers, and Taq polymerase (Applies Biosystems, Foster City, CA, USA). The primer sequences were as follows: forward 5′-tacccacgtatgtaccaccc-3′ and reverse 5′-gcactgttctgatcacgtcg-3′. PCR conditions were denaturation at 95°C for 10 min, 40 cycles of 94°C for 30 s, 58°C for 30 s, and 72°C for 30 s, and final extension at 72°C for 7 min. After purification using calf intestine alkaline phosphatase (CIP, Promega, Madison, WI, USA), 1.0 *µ*L of purified PCR product was mixed with 2.5 *µ*L of SNaPshot ready Reaction Mix (ABI) and 20 pmol/*µ*L of SNaPshot primer (5′-aagagctcttttgtctttca-3′). The cycling program was 25 cycles consisting of 96°C for 10 s, 50°C for 5 s, and 60°C for 30 s. Postextension products were purified with CIP and incubated at 37°C for 45 min and at 75°C for 10 min. Then, 1 *µ*L of the final reaction samples containing the extension products was added to 9 *μ*L Hi-Di formamide (Applied Biosystems). The mixture was incubated at 95°C for 5 min, followed by 5 min on ice, and then analyzed by electrophoresis on an ABI Prism 3730 DNA analyzer. Results were analyzed using GeneScan Analysis 3.1 (Applied Biosystems) software. The CYP3A5 A6986G (rs776746) polymorphism was analyzed for the detection of the *3 allele, since previous reports suggested that CYP3A5*3 is the major defective allele and that other functional exonic SNPs are rare in the Japanese population [13]. With regard to the CYP3A5 genotype, patients were allocated into 2 groups: CYP3A5*1/*1 or CYP3A5*1/*3 (expressors) and CYP3A5*3/*3 (nonexpressors).

### 2.6. Statistical Analyses

All values were expressed as the mean ± standard deviation (SD) and median as appropriate. Fisher's exact tests were used for categorical factors. Student's *t*-test was used to compare ATP levels and tacrolimus C/D ratio between infection versus no infection, Hep C recurrence versus no recurrence, and rejection versus no rejection. The Pearson correlation coefficient was used to determine the relationship between the blood concentration of tacrolimus and the dosage of tacrolimus, and between the blood concentration of tacrolimus and the IMK ATP levels. Data were analyzed using statistics computer software Pharmaco Analyst II (Hakuhousha Co., Tokyo, Japan). A *P* value <0.05 was considered to indicate a statistically significant difference.

## 3. Results

### 3.1. Pharmacokinetics of Tacrolimus and IMK ATP Level

**Figure 1:**
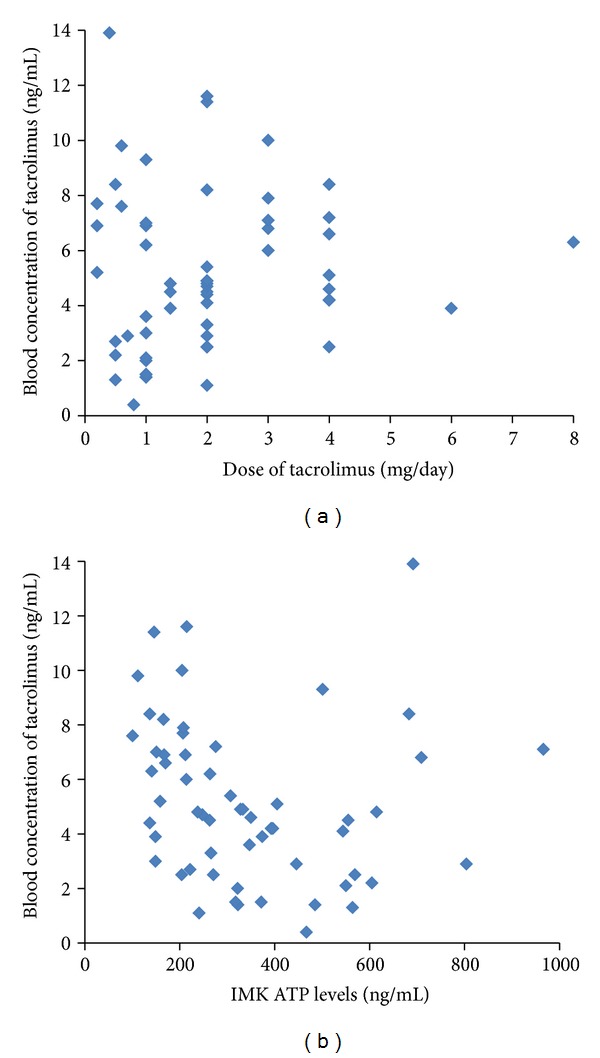
(a) Relationship between the blood concentration of tacrolimus and dosage of tacrolimus after LDLT (*R* = 0.0806, *P* = 0.1162). (b) Relationship between blood concentration of tacrolimus and ImmuKnow ATP level after LDLT (*R* = 0.1473, *P* = 0.2745).

The blood concentration of tacrolimus ranged from 0.4 to 13.9 ng/mL, and the dosage of tacrolimus ranged from 0.4 to 8.0 mg/day in each recipient. There was no statistically significant relationship between the blood concentrations of tacrolimus and the dosage of tacrolimus in LDLT recipients (*R* = 0.0806, *P* = 0.1162) (Figure 1(a)). There was also no statistically significant relationship between the blood concentrations of tacrolimus and the ATP levels in LDLT recipients (*R* = 0.1473, *P* = 0.2745) (Figure 1(b)). Clinically, there were no samples that behave like outliers in both figures.

### 3.2. IMK ATP Level and Tacrolimus C/D Ratio in Patients with or without Infection

**Figure 2:**
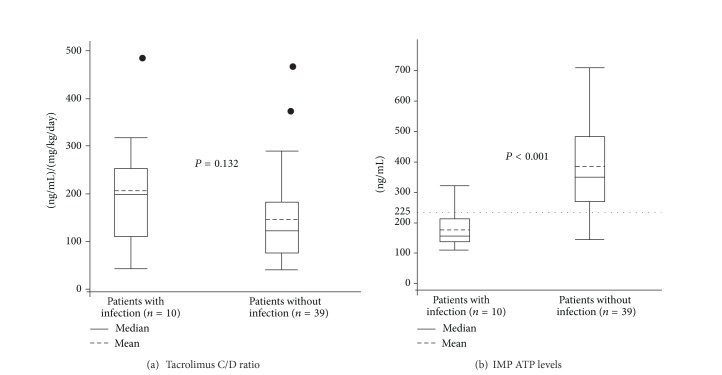
IMK ATP levels and tacrolimus C/D ratio in patients with or without infection. (a) The ATP was 218.4 ± 129.2 (range 112–312) ng/mL and 149.3 ± 99.1 (range 146–706) ng/mL, respectively. (b) The ATP was 185.5 (range 112–312) ng/mL and 350 (range 146–706) ng/mL, respectively.

Posttransplant bacterial and viral infection occurred in 10 of 49 patients (20.4%). The mean tacrolimus C/D ratios were 218.4 ± 129.2 ng/mL per mg/kg/day in patients with infection and 149.3 ± 99.1 in patients without infection. There was no significant difference between two groups (*P* = 0.132) (Figure 2(a)). The mean ATP levels in patients with infection (*n* = 10) was significantly lower than that in patients without infection (*n* = 39) (185.5 ± 64.5 ng/mL versus 350 ± 159.7 ng/mL, *P* < 0.001) (Figure 2(b)).

### 3.3. IMK ATP Level and Tacrolimus C/D Ratio in Patients with or without Rejection

**Figure 3:**
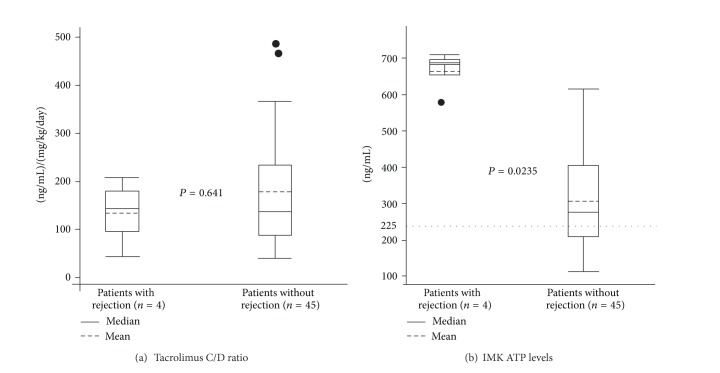
IMK ATP levels and tacrolimus C/D ratio in patients with or without rejection. (a) 134.1 ± 71.9 (range 112–312) ng/mL and 179.2 ± 133.6. (b) The ATP was 663.2 (range 569–709) ng/mL and 306.6 (range 146–615) ng/mL, respectively.

Histologically proven rejection occurred in 4 cases (8.2%). The mean tacrolimus C/D ratios were 134.1 ± 71.9 ng/mL per mg/kg/day in patients with rejection (*n* = 4) and 179.2 ± 133.6 in patients without rejection (*n* = 45), showing no significant difference between two groups (*P* = 0.641) (Figure 3(a)). The mean ATP levels in patients with rejection was significantly higher than that in patients without rejection (663.2 ± 63.6 ng/mL versus 306.6 ± 138.7 ng/mL, *P* < 0.001) (Figure 3(b)).

### 3.4. IMK ATP Level and Tacrolimus C/D Ratio in Patients with Hepatitis C

**Figure 4:**
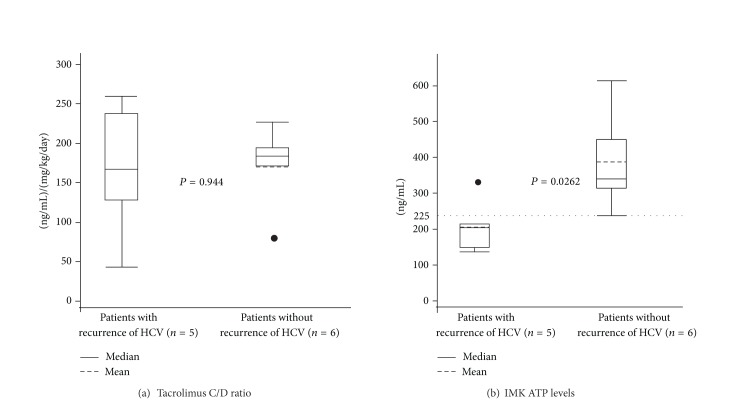
IMK ATP levels and tacrolimus C/D ratio in patients with hepatitis C. (a) 166.4 ± 94.2 (range 112–312) ng/mL and 170.4 ± 56.8. (b) The ATP was 215.0 (range 141–322) ng/mL and 398.4 (range 238–615) ng/mL, respectively.

Histologically proven recurrence of hepatitis C occurred in 5 cases (45.5%) out of 11 patients with hepatitis C. The mean tacrolimus C/D ratios were 166.4 ± 94.2 ng/mL per mg/kg/day in patients with recurrence (*n* = 5) and 170.4 ± 56.8 in patients without recurrence (*n* = 6). There was no significant difference between two groups (*P* = 0.944) (Figure 4(a)). The mean ATP levels in patients with recurrence of hepatitis C was significantly lower than that in patients without recurrence (205.6 ± 73.4 ng/mL versus 387.7 ± 137.5 ng/mL, *P* = 0.0262) (Figure 4(b)).

### 3.5. IMK ATP Level in the Patients Who Developed Special Clinical Events

During this survey, 14 of the 49 patients experienced special clinical events, such as bacterial infectious complications, recurrence of hepatitis C (RHC), and acute cellular rejection (ACR), as shown in Table 2. All of the patients suffered from bacterial infectious complications and 4 out of 5 patients who developed RHC showed ATP levels lower than 225 ng/mL. On the other hand, the ATP levels in all patients with ACR were higher than 525 ng/mL.

When we used cut-off ATP level of 225 ng/mL for identifying risk of infection and 525 ng/mL for rejection according to a previous report [8], diagnostic accuracy of IMK for identifying risk of infection was favorable with sensitivity of 0.909 and specificity of 0.842, and that of rejection was also satisfactory with sensitivity of 1.0 and specificity of 0.911.

### 3.6. Influence of CYP3A5 Genotype on the Incidences of Postoperative Infectious Complication and Acute Cellular Rejection

Table 3 shows the relationship between the patients with infectious complication or acute rejection and those divided by CYP3A5 genotype. The incidence rate of infectious complications did not differ among 4 groups. Additionally, incidence of acute cellular rejection was also similar among 4 groups.

## 4. Discussion

One of the most important things to improve long-term graft survival is the maintenance of an adequate level of immunosuppressive agents. Neuberger [13] analyzed the common causes of death in 617 patients more than five years after transplantation and concluded that cardiovascular disease (22%), *de novo* malignancy (19%), infection (19%), and chronic rejection (5%) were responsible for the majority of deaths among them, which means that the major causes of late death were immunosuppression-related diseases. 

The IMK assay is considered as a useful tool of monitoring of immune activity in transplant recipients; however, the predictive capability of IMK for identification of infection and rejection during the late period after LT remains unclear. Till now, there are two studies of meta-analysis regarding IMK. Rodrigo et al. [14] reported that the IMK is a valid tool for determining the risk of further infection in LT recipients, but the ability of IMK for detecting of rejection was controversial because of significant heterogeneity across studies. Ling et al. [15] suggest that IMK is not able to identify individuals at risk of infection or rejection and additional studies are still needed to clarify the usefulness of this test. However, regarding the individual studies included in these meta-analyses, IMK was measured at different time points after transplantation. In this present study, we focused on late allograft dysfunction, especially in patients more than one year after LT.

In our study, we had five bacterial infectious disease cases, five recurrent HCV cases, and four patients with rejection during the period of our surveillance, and almost all of these patients showed the IMK ATP levels out of the target immunological response zone, suggesting the usefulness of this test for identifying risks of infection and rejection. Fortunately, none of them were lethal, and bacterial infections were cured by administration of the antibiotics and rejections were successfully treated by steroid pulse and increase of dosage of immunosuppressive agents. In LT patients with HCV, we have to decide very carefully the adequate level of immunosuppressive agents because the use of strong immunosuppression during the treatment of acute rejection causes early and severe recurrence of HCV in the post-LT setting [16–18].

Recurrence of HCV in LT leads to cirrhosis in 30% of the patients within 5 years following LT [19], and 41% have severe fibrosis or cirrhosis 6 years after LT [20], so our transplant physicians have to reduce immunosuppressive agents as much as possible. Although liver biopsy is considered to be the gold standard for assessing fibrosis progression of HCV, it is an invasive procedure with some limitations, such as coagulopathy and thrombocytopenia. Several authors reported that the IMK assay can be a helpful tool to monitor the immune status in LT patients with HCV: HCV-positive recipients with recurrent disease had significantly lower ATP levels than those without recurrence [21], the sensitivity and specificity for distinguishing recurrent HCV from acute cellular rejection were 88.5% and 90.9% [22], and the progression of fibrosis in HCV-positive recipients has been correlated with lower IMK ATP levels [23]. We also suggested the IMK assay reflected the immunological aspects of the LT patients with HCV; however, the small sample size and lack of a validation group of patients should be taken into consideration.

CYP3A5 is known to play a key role in the pharmacokinetics of tacrolimus in LT patients and to be responsible for the interindividual variations in the pharmacokinetics of tacrolimus in the recipients until 12 months after LDLT [24]. We revealed that the tacrolimus C/D ratio was significantly lower in subjects with CYP3A5*1 alleles (expressor) than in those with the CYP3A5*3 allele (nonexpressor), suggesting that high dose of tacrolimus is required in expressors to achieve the target trough levels [7]. We also previously suggested that there was higher risk for the development of infectious complications in patients with the CYP3A5*1 allele (expressor) than in those with the CYP3A5*3 allele (nonexpressor) until 6 months after LDLT, because the patients with expressors tend to be overimmunosuppressed [10]. However, our study suggested that there was no association between CYP3A5 and infectious complication or acute rejection in patients more than one year after LDLT. These results suggest that CYP3A5 in both recipient and donor mainly affects tacrolimus pharmacokinetics during early postoperative period but not late phase after operation.

Our study has some limitations. The IMK assays were undergone at some haphazard times and no clear reasons for this assay could be identified, which means either inadequate ancillary studies or questionable indications for the IMK assays. The small number of allograft rejection episodes rendered the analysis of this subgroup very difficult. In addition, the CYP3A5 genotypes were examined in only 39 patients (79.5%). Although our results are interesting, their broad applicability in other transplantation centers, in which immunosuppression and management protocols are different, should be justified as a part of future studies, because of the small sample size, its retrospective nature, and the use of single time point measurements of IMK ATP levels in some patients.

## 5. Conclusion

In conclusion, our study identifies the IMK assay as a useful tool for monitoring immunological aspects on late allograft dysfunction, including bacterial infection, recurrence of HCV, and ACR. CYP3A5 in both recipient and donor did not affect tacrolimus pharmacokinetics during late phase after operation.

## Figures and Tables

**Table 1:** Characteristics of the 49 patients.

Age	51 (20–69)
Male/female	33/16
Etiology of LDLT	
HCV (HCC)	11 (6)
HBV (HCC)	11 (3)
NBNC (HCC)	10 (1)
PBC	6
Others	11

Median months after LDLT	54.6 (12.2–111.5)

Laboratory data	
AST (U/L)	41.4 (14–257)
TB (mg/dL)	1.5 (0.3–3.7)
WBC (mm^−3^)	6161 (2480–10480)

Dose of tacrolimus (mg/day)	2.3 (0.5–8)
Blood concentration of tacrolimus (ng/mL)	5.4 (0.4–10.3)
C/D ratio (ng/mL per mg/kg/day)	170.3 (40.3–599.5)

LDLT: living donor liver transplantation, HCV: hepatitis C virus, HCC: hepatocellular carcinoma, HBV: hepatitis B virus, PBC: primary biliary cirrhosis, LC: liver cirrhosis, FH: fulminant hepatitis, ALT: alanine aminotransferase, TB: total bilirubin, WBC: white blood cell count, C/D ratio: tacrolimus concentration/dose ratio.

**Table 2:** IMK ATP levels in the patients who experienced late clinical events.

Age	Gender	Etiology of LDLT	Clinical events	Months after LDLT	Tacrolimus concentration (ng/mL)	C/D ratio (ng/mL/mg/kg/day)	IMK ATP levels (ng/mL)
46	F	LC	Phlegmon	89.5	9.8	99.76	112
60	M	LC	Cholangitis	103.3	8.4	478.80	113
58	M	HCV, HCC	RHC	57.9	6.3	43.31	137
20	F	PSC	Cholangitis	92.1	3.9	105.86	141
60	M	HCV	RHC	27.1	8.2	259.12	149
61	F	LC, HCC	UTI	34.9	10	229.00	166
68	M	HCV, HCC	RHC	52.4	7.9	166.95	205
62	F	HCV, HCC	RHC	86.3	8.6	237.79	215
52	M	PBC	Pneumonia	55.5	2.7	316.44	222
50	M	HCV	RHC	17.3	2.0	128.80	322

51	M	PBC	Rejection	38.8	2.5	43.25	569
43	M	FH	Rejection	45.4	8.4	171.57	683
58	F	HBV, HCC	Rejection	19.9	9.9	207.90	692
25	M	BA	Rejection	104.7	6.8	113.33	709

IMK: ImmuKnow, LDLT: living donor liver transplantation, C/D ratio: concentration/dosage ratio, LC: liver cirrhosis, HCV: hepatitis C virus, HCC: hepatocellular carcinoma, PSC: primary sclerosing cholangitis, PBC: primary biliary cirrhosis, HBV: hepatitis B virus, FH: fulminant hepatitis, BA: biliary atresia, RHC: recurrence of hepatitis C, UTI: urinary tract infection.

**Table 3:** Relationship between the patients with infectious complication or acute rejection and those divided by CYP3A5 genotype.

	EX-R/EX-D (*n* = 5)	EX-R/NEX-D (*n* = 6)	NEX-R/EX-D (*n* = 10)	NEX-R/NEX-D (*n* = 18)	Value
Infection	0	1 (16.7%)	3 (30%)	5 (27.7%)	n.s.
Rejection	1 (25%)	1 (16.7%)	1 (10%)	1 (5.5%)	n.s.

EX: expressor (*1/*1 or *1/*3), NEX: nonexpressor (*3/*3).

EX-R: recipient with EX, NEX-R: recipient with NEX.

EX-D: donor with EX, NEX-D: donor with NEX.

n.s.: nonsignificant.

## Abbreviations

IMK: ImmuKnow immune cell function assay
CYP3A5: Cytochrome P450 3A5
LDLT: Living donor liver transplantation
LT: Liver transplantation
C/D ratio: Concentration/dose ratio.
